# Sensitive and rapid RT-RPA-Cas12a-mediated detection method capable of human rhinovirus A and/or C species by targeting VP4

**DOI:** 10.1016/j.virusres.2022.199001

**Published:** 2022-11-12

**Authors:** Weidong Qian, Xuefei Wang, Jie Huang, Jian Liu, Si Chen, Ting Wang, Dandan Zhang, Yongdong Li

**Affiliations:** a School of Food and Biological Engineering, Shaanxi University of Science and Technology, Xi'an 710021, PR China; b Shanghai Animal Disease Prevention and Control Center, Shanghai 201103, PR China; c University of Shenzhen, Shenzhen 518052, PR China; d Ningbo Municipal Center for Disease Control and Prevention, Ningbo 315010, PR China

**Keywords:** Human rhinovirus, Reverse-transcription recombinase polymerase amplification, CRISPR-Cas12a, Detection, Rhinovirus species A or C

## Abstract

Human rhinovirus (HRV), the main etiologic agent of the common cold, is responsible for significant morbidity, medical costs, and the loss of productivity in the workplace and school. To prevent the spread of HRV, accurate, low-cost and rapid diagnostics of HRV is crucial for identifying those at-risk for the illness associated with HRV, with the most frequently detected species, including HRV species A (HRV-A) and C (HRV-C). Here, a novel HRV-A and/or HRV-C molecular diagnostic assay that integrates reverse-transcription recombinase polymerase amplification assay (RT-RPA) amplification with CRISPR/Cas12a detection, with the result readout using a fluorescence detector or lateral flow strip (LFS). The established assay could be completed within 50 min without complex instruments and skilled technicians. The limit of detection of the RT-RPA-Cas12a-mediated real-time fluorescence or LFS assay could reach 0.1 copy/μl, and 0.5 copy/μl for the end-point fluorescence assay with a UV light illuminator readout, respectively. Meanwhile, the assay demonstrates excellent specificity without cross-reactivity to non-target viruses. Furthermore, they were appraised using 80 clinical samples, and RT-RPA-Cas12a-mediated fluorescence or LFS assay displayed high-accuracy with positive and negative predictive agreement of 96.7%, 95% and 100%, respectively. Taken together, the RT-RPA-Cas12a-mediated assay is a rapid, sensitive, and specific detection tool for routine and on-site detection method for HRV-A and/or HRV-C infections, and shows great promise for use in resource-poor or constrained settings.

## Highlights

• The fluorescence or LFS assay based on RT-RPA-Cas12a is a point-of-care diagnosis method.
• The fluorescence or LFS assay based on RT-RPA-Cas12a detection can be completed within 50 min with high specificity.
• The RT-RPA-Cas12a mediated fluorescence or LFS assay demonstrates high sensitivity (0.1 or 0.5 copy per microliter).

## 1 Introduction

Since the discovery of human rhinovirus (HRV) in the 1950s, HRV has prevailed across the world, and has been the leading causes of upper respiratory tract infections (Jacobs et al., 2013). Individuals with HRV infection can experience a variety of clinical manifestations, ranging from common colds (Hayden, 2004), otitis media, and sinusitis (Jacobs et al., 2013; Winther, 2011; Kiang et al., 2007; Henquell et al., 2012) to critical illness such as bronchiolitis or pneumonia (Jacobs et al., 2013; Ison et al., 2003). Further, with the effective and applicable diagnostic methods available since the 2000s, HRV has been also established as the causative agent of severe pneumonia in the elderly or immunocompromised individuals, as well as exacerbations of chronic obstructive pulmonary disease and asthma (Friedlander and Busse, 2005; McManus et al., 2008; Leigh and Proud, 2015; Kurai et al., 2013).

HRV is the single-stranded, positive-sense and non-enveloped RNA virus belonging to the genus Enterovirus in the family of Picornaviridae (Golke et al., 2021). Until recently, there are more than 160 different genotypes of HRV, which can be mainly divided into three categories: HRV-A, HRV-B, and HRV-C species (http://www.picornaviridae.com/enterovirus/enterovirus.htm). Among 160 different genotypes, more than classical 100 genotypes belonging to HRV-A or HRV-B species have been recognized since the 1950s, while until 2006 HRV-C species including approximately 50 genotypes, was firstly found, since it cannot be propagated *in vitro* under standard cell culture conditions (Lamson et al., 2006; Giardina et al., 2022). Currently, the most frequently detected species are HRV-A and HRV-C (Giardina et al., 2022), and the HRV-C species have frequently been detected among children and have resulted in acute respiratory infection in children (Watanabe et al., 2010). In contrast, the HRV-B species represent the less frequently detected (Giardina et al., 2022). Though HRV associated with diseases represent an important socioeconomic burden worldwide, no approved antivirals or vaccines for the treatment of HRV currently exist (Jia et al., 2022).

The focus of prevention and control of infectious diseases caused by HRV is currently still in the early detection on site and outbreak control stages, which can significantly boost the quality of the management and treatment of HRV-related diseases, and reduce antibiotic misuse (Qin et al., 2022). Therefore, accurate, efficient, and low-cost diagnosis methods are required in the routine, on-site diagnostics or wide-scale population screening use. The diversity of HRV serotypes has hampered the exploitation of antibodies-based serological detection methods (Milanoi et al., 2016). Typically, quantitative real-time reverse-transcription PCR (qRT-PCR) is considered as the sensitive method for the molecular diagnosis of HRV. Unfortunately, there are several limitations of qRT-PCR, including the need of expensive and sophisticated instruments and trained professionals for data processing as well as difficult to implement in some regions, which constitutes a challenge in limited resource availability and limit its application for point-of-care testing (POCT). To address these limitations, various attempts have been undertaken, including the development of nucleic acid amplification methods without complex instruments. Isothermal amplification techniques based on the relatively low temperature, such as reverse-transcription recombinase polymerase amplification (RT-RPA) (Li et al., 2018; Garrido-Maestu and Prado, 2022), loop-mediated isothermal amplification (LAMP) (Owoicho et al., 2022), cross-priming amplification (CPA) (Bates and Zumla, 2016), and reverse-transcription strand invasion-based amplification (RT-SIBA) (Zhang et al., 2021), have been developed for the detection of viral pathogens without costly specialized equipment or highly trained staff, enabling them ideal tools to support community surveillance or on-site detection in resource-constrained setting. Nevertheless, false-negative results could arise due to the low-temperature enzyme-based DNA amplification strategy (Qin et al., 2022; Tomita et al., 2008; Wang et al., 2017), and the diagnostic accuracy needs to be further improved.

A promising novel nucleic acid sensing platform is clustered regularly interspaced short palindromic repeat (CRISPR)-associated endonuclease (CRISPR/Cas) system-based method, in which Cas effector proteins (*e.g.*, Cas9, Cas12a, Cas12b, Cas13a and Cas14), are applied due to their highly specific sequence recognition and the collateral cleavage activity (Yan et al., 2019; Abudayyeh et al., 2016; Gootenberg et al., 2018; Harrington et al., 2018). Particularly, Cas12a (formerly Cpf1), an RNA-guided Class II type V-A CRISPR nuclease guided by a programmable CRISPR RNA (crRNA), only requires a short crRNA to perfectly form a complementary sequence with the target DNA at physiological temperature or even at room temperature (Chen et al., 2018). Upon formation of the Cas12a-crRNA-target DNA complex, Cas12a is activated and then exhibits a robust collateral cleavage activity to indiscriminately cleave surrounding nonspecific, non-target single-stranded DNA (ssDNA). Meanwhile, the Cas12a-mediated non-specific, trans cleavage is multiple turnover, thereby enabling the accurate and specific detection of low abundance targets (Chen et al., 2018). By combining fluorescent-based probe or lateral flow assay (LFS), many CRISPR-Cas12a-based diagnostic methods have been extensively developed for the detection of various pathogens (Mohammad et al., 2022; Qian et al., 2021a).

**Fig. 1:**
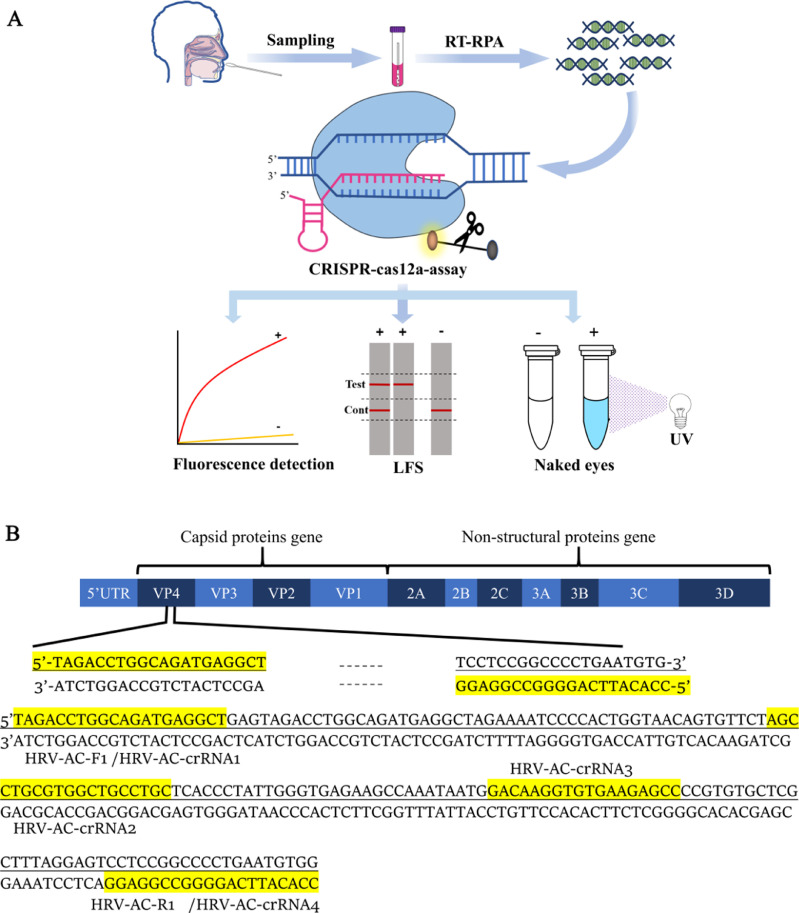
(A) Schematic diagram of RT-RPA-Cas12a-mediated real-time and end-point fluorescence, and LFS assay with readouts using the fluorescence reporter, including FAM/BHQ1 labeled ssDNA (ssDNA FQ) or FAM/Biotin labeled ssDNA (ssDNA FB). ssDNA FQ was employed and cleaved for the RT-RPA-Cas12a-mediated real-time and end-point fluorescence assay. Under the guidance of crRNA, Cas12a recognizes target DNA by RT-RPA, forms a ternary complex of Cas12a, crRNA, and target DNA, and then trans-cleaves ssDNA FQ. Then, the fluorescence signal of FAM is restored, and can be easily and inexpensively detected using a fluorescence reader, or by the naked eyes under a UV light illuminator. In contrast, ssDNA FB was employed and cleaved for the RT-RPA-Cas12a-mediated LFS assay. After ssDNA FB is cleaved by the activated Cas12a, the dissociated FAM and Biotin is visualized on a LFS. The whole process can be completed within 50 min using a fluorescence reader or LFS. RT-RPA, reverse-transcription recombinase polymerase amplification assay; Test, test band; Cont, control band. (B) Schematic representation of human rhinovirus genome encoding a polyprotein. Four structural (VP1, VP2, VP3, VP4) and other non-structural proteins are indicated. The target consensus sequence of VP4 for rhinovirus A and C species is shown.

Based on their wide distribution and associated medical consequences, as well as the lack of POCT diagnostic method of HRV-A and HRV-C in under-resourced health care settings, we developed RT-RPA-Cas12a-mediated fluorescence or LFS assays for rapid diagnosis of HRV-A and/or HRV-C, which can be conducted at 39 °C in only 40–50 min without complex equipment. The test results can be obtained based on fluorescence intensity and visual examination analysis under low cost conditions. In this study, the optimized RT-RPA amplification and CRISPR/Cas12a detection system were combined, with the real-time or end-point readout using a fluorescence detector or LFS (Fig. 1A). Our established RT-RPA-Cas12a-mediated fluorescence or LFS assay is a rapid, sensitive, and specific detection tool for routine and on-site detection method for HRV-A and/or HRV-C infections, and shows great promise for use in resource-poor or constrained settings.

## 2 Materials and methods

### 2.1 Clinical samples

A total of 80 nasopharyngeal aspirates were obtained from patients with lower respiratory tract infection and diagnosed as HRV-A- (32 clinical samples), HRV-B- (10 clinical samples), or HRV-C-positive (28 clinical samples) with Ct values under 30 (< 30 Ct) according to the previously described method (Wisdom et al., 2009), and 10 viral samples from respiratory tract defined as HRV-A-or HRV-C-negative, including metapneumovirus (HmPV), adenovirus (ADV), bocavirus (HBoV), and respiratory syncytial virus (RSV), were employed as the control. All specimens were collected by Ningbo Municipal Center for Disease Control and Prevention (NCDC) between January 2018 and December 2020. This study was approved by the ethical committee of NCDC.

### 2.2 Reagents

ssDNA reporters, including FAM/BHQ1 labeled ssDNA (ssDNA FQ) for fluorescence readout and FAM/Biotin labeled ssDNA (ssDNA FB) for LFS readout, were manufactured by GENEWIZ Inc. (Suzhou, China). Oligonucleotides of all the primers for RT-RPA and crRNA in this study were produced by Sangon Biotech (Shanghai, China), and presented in Table 1. RT-RPA kit was bought from Jiangsu lesun biotechnology Co., Ltd (Wuxi, China). Cas12a and its reaction buffer NEB buffer 2.1 were purchased from New England Biolabs (MA, USA), and the LFS was purchased from Tiosbio (Nanjing, China).

**Table 1:** Oligonucleotides employed for RT-RPA and crRNA in RT-RPA-Cas12a-mediated assay for rhinovirus species A or C detection.

Assay	Name	Oligonucleotide sequences (5′−3′)

RT-RPA	HRV-A/C-F	TAGACCTGGCAGATGAGGCT
	HRV-A/C-R1	CACATTCAGGGGCCGGAGGA
	HRV-A/C-R2	ACGGACACCCAAAGTAGTTGGTTCCATCCC
Cas12-based detection	crRNA1-F	GAAATTAATACGACTCACTATAGGGTAATTTCTACTAAGTGTAGATTAGACCTGGCAGATGAGGCT
	crRNA1-R	AGCCTCATCTGCCAGGTCTAATCTACACTTAGTAGAAATTACCCTATAGTGAGTCGTATTAATTTC
	crRNA2-F	GAAATTAATACGACTCACTATAGGGTAATTTCTACTAAGTGTAGATAGCCTGCGTGGCTGCCTGC
	crRNA2-R	GCAGGCAGCCACGCAGGCTATCTACACTTAGTAGAAATTACCCTATAGTGAGTCGTATTAATTTC
	CrRNA3-F	GAAATTAATACGACTCACTATAGGGTAATTTCTACTAAGTGTAGATGACAAGGTGTGAAGAGCC
	CrRNA3-R	GGCTCTTCACACCTTGTCATCTACACTTAGTAGAAATTACCCTATAGTGAGTCGTATTAATTTC
	CrRNA4-F	GAAATTAATACGACTCACTATAGGGTAATTTCTACTAAGTGTAGATTCCTCCGGCCCCTGAATGTG
	CrRNA4-R	CACATTCAGGGGCCGGAGGAATCTACACTTAGTAGAAATTACCCTATAGTGAGTCGTATTAATTTC
	ssDNA FQ ssDNA FB	6-FAM-TTATTATT-BHQ1 6-FAM-TTATTATT-Biotin

### 2.3 Design of primers for RT-RPA and crRNA, as well as preparation of crRNA

Based on the consensus sequences for HRV-A and HRV-C, both two pairs of RT-RPA primers and four pairs of oligonucleotides for preparation of crRNA were designed, followed by the synthesis by Suzhou GENEWIZ biotech, and shown in Table 1. The primer specificity was checked by nucleotide BLAST (blastn), and the secondary structure and dimer analysis were performed using Oligo Analyzer 3.1. Preparation of crRNA was mainly conducted using the following two-step. Four pairs of oligonucleotides were annealed to generate double strand DNAs (dsDNA), respectively, and then the resulting dsDNA products were transcribed by *in vitro* transcription (IVT) using IVT T7 Kit. The *in vitro* transcription reaction was performed by incubating the mixture, including 5 μL of 10× transcription buffer, 5 μL of each NTP solution, 1 μL of RNase inhibitor, 5 μL of T7 RNA polymerase, 9 μL of RNase-free water and 10 μL of annealed dsDNA, at 42 °C for 2 h. The resulting crRNA products were subjected to phenol/chloroform extraction, and the amount of crRNA was measured using the spectrophotometer (Metash Instruments, Shanghai, China).

### 2.4 Preparation of standard RNA of the consensus sequences for HRV-A and HRV-C

30 published full-genome sequences of HRV-A and HRV-C submitted to GenBank, including MH648096.1, JX177627.1, MN749154.1, FJ445177.1, KM109980.1, OM001459.1, JN837688.1, DQ875932.2, JN990702.1, JQ837720.1, JX074056.1, JQ245968.2, MZ629144.1, OK017914.1, KF734978.1, KJ675505.1, KP890662.1, MZ438010.1, NC_009996.1, EF582387.1, EF582386.1, JF317017.1, GQ223228.1, JN205461.1, GQ323774.1, KX398052.1, JF781504.1, JF285323.1, JN798567.1, HQ123440.1, were retrieved and aligned using the MEGA X. Sequence comparison analysis of HRV-A and HRV-C full-genome revealed that a fraction within the VP4 region ranging from 188 bp to 578 bp is highly conserved, and was identified as the target (Fig. 1B). Then the conserved DNA fragment of approximately 392 nucleotides was synthesized by Shanghai Sangon biotech (Shanghai, China), and integrated into the pBluescript II SK (+) to construct pBluescript-VP4. Subsequently, the resulting pBluscript-VP4 was transformed into *Escherichia coli* TOP10 to prepare recombinant strains harboring the pBluescript-VP4, and stored at −80 °C.

To prepare standard RNA of the consensus sequences for HRV-A and HRV-C, the pBluescript-VP4 was extracted and purified using the TIANprep Mini Plasmid Kit (Tiangen Biotech, Beijing, China), and further cut with *Sac* I. The resulting linearized pBluescript-VP4 was applied as the template of the IVT reaction to produce standard RNA of the consensus sequences for HRV-A and HRV-C using IVT T7 Kit (TaKaRa, Dalian, China). Briefly, the IVT reaction mixture, including 5 μL of 10× transcription buffer, 5 μL of each NTP solution, 1 μL of RNase inhibitor, 5 μL of T7 RNA polymerase, 6.5 μL of RNase-free water and 12.5 μL of linear pBluescript-VP4 plasmid, was incubated at 42 °C for 2 h. Eventually, the number copies of RNA were determined using the formula: RNA number of copies = (amount of RNA in nanograms ×6.023 × 10^23^) / (length of RNA in base pairs ×10^9^ × 330).

### 2.5 RT-RPA-Cas12a-mediated fluorescent assay

The RT-RPA-Cas12a-mediated fluorescent assay is mainly divided into two steps (Fig. 1). The first step of this assay involves the exponential amplification of the target sequence by RT-RPA. The 50 μL RT-RPA reaction mixture was performed in the preheated Axxin T8 isothermal instrument for 20 min at 39 °C, including 25 μL of reaction buffer V, 2 μL of each primer (10 μM), 13.5 μL of ddH_2_O, 5 μL of standard RNA and 2.5 μL of 280 mM magnesium acetate. For the negative control, RNase-free water was served as the template in the same volume. At the second stage, the Cas12a-mediated trans-cleavage reaction was carried out in a 50 μL reaction mixture, including 5 μL of 10 × NEB Buffer 2.1, 2 μL of ssDNA FQ or ssDNA FB (1 μM), 1 μL of 2 μM Cas12a, 4 μL of 1 μM crRNA, 0.5 μL RNase inhibitor (40 U), 27.5 μL of deionized water, and 10 μL of RT-RPA products or deionized water (the negative control). The Cas12a-mediated reaction was performed at 37 °C for 20 min. The detection results of the assay were obtained with fluorescent signals collected every 10 s, or visualized under a UV light illuminator with ssDNA FQ as the substrates, and by LFS with ssDNA FB as the substrates.

### 2.6 Optimization of crRNA and the reaction concentration for RT-RPA-Cas12a-mediated fluorescent or LFS assay

The Cas12a-mediated trans-cleavage reaction was examined, following the conditions as described above. To screen the optimal crRNA, a single or combination of crRNA was employed, and the detection result was assessed by the intensity of fluorescent signal. Then, to determine the optimal concentration of crRNA for the trans-cleavage activities mediated by Cas12a, final concentrations ranging from 0 nM to 120 nM were used in the RT-RPA-Cas12a-mediated fluorescent assay. Afterwards, the Cas12a-mediated trans-cleavage activities were examined in the presence of the optimal crRNA at various concentrations (0, 100, 200, 400 and 600 nM) in the RT-RPA-Cas12a-mediated LFS assay.

### 2.7 Specificity of RT-RPA-Cas12a-mediated fluorescent or LFS assay

The specificity of RT-RPA-Cas12a-mediated fluorescent or LFS assay was conducted by evaluating various RNA or DNA samples from other respiratory viruses at the final concentration of 200 copies/μL, including HRV-B, HmPV, ADV, HBoV, and RSV. 10 different samples tested positive for each test virus, and 10 HRV-B samples were applied in this assay.

### 2.8 Sensitivity of RT-RPA-Cas12a-mediated fluorescent or LFS assay

To examine the sensitivity of RT-RPA-Cas12a-mediated fluorescent or LFS assay, a concentration gradient of RNA standard samples (1000, 100, 10, 1, 0.5, 0.1, 0.05 and 0 copies/µL), was employed as the template in RT-RPA-Cas12a-mediated reaction. Each reaction was replicated three times and the detection results were analyzed by real-time and endpoint fluorescence, as well as LFS.

### 2.9 qRT-PCR for clinical samples

The total RNAs of 60 HRV-A or HRV-C samples mentioned in Section 2.1 were manually extracted using BeaverBeads™ Viral DNA/RNA Kit BEAVER, Suzhou, China), and automatically conducted using automatic nucleic acid extraction instrument (bioPerfectus technologies, Jiangsu, China), respectively. The qRT-PCR detection for HRV nucleic acids was conducted using HRV kit (bioPerfectus technologies, Jiangsu, China) in an ABI 7500 (Applied Biosystems). The reactions were carried out with first step of reverse transcription at 42 °C for 30 min, followed by the reaction parameters: 95 °C for 10 min, 45 cycles of 95 °C for 30 s, 55 °C for 30 s and 72 °C for 30 s.

### 2.10 Statistics

Each sample was performed in at least three independent biological replicates. Statistical analysis of the end-point fluorescence value was conducted using the GraphPad Prism 8 (GraphPad Software, version 8.0.1), and statistical significance was determined by the Students' *t*-test.

## 3 Results

### 3.1 Establishment of the RT-RPA-Cas12a-mediated fluorescence assay

**Fig. 2:**
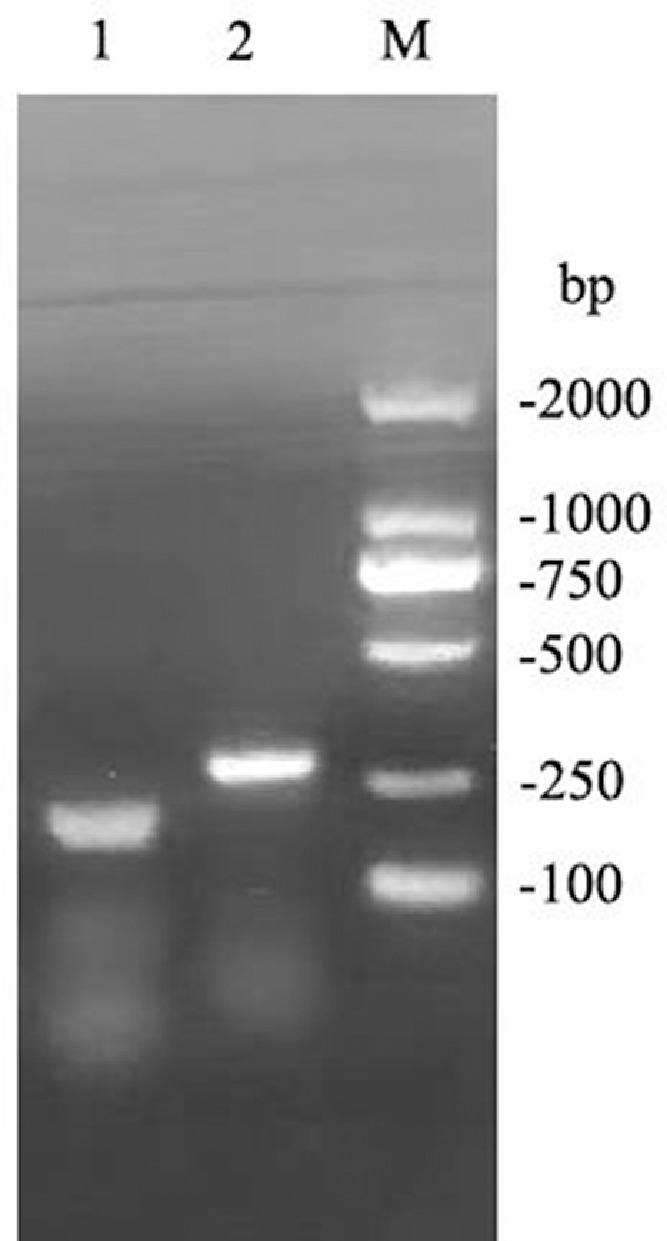
Evaluation of the performance of RT-RPA primers using agarose gel electrophoresis. M, DL2000 DNA marker; 1–2, RT-RPA products amplified with HRVA/C-F/HRVA/C-R1 and HRVA/C-F/HRVA/C-R2, respectively.

In order to examine the performance of RT-RPA primers, RT-RPA assays were performed using the final concentration of 1 × 10^5^ copies/μL of standard RNA of the consensus sequences for HRV-A and HRV-C as a template, and two pairs of primers, including HRVA/C-F/HRVA/C-R1 and HRVA/C-F/HRVA/C-R2. The reaction was conducted at 39 °C for 20 min using RT-RPA kit. In contrast to HRVA/C-F/HRVA/C-R1, HRVA/C-F/HRVA/C-R2 was capable of amplifying a clear single and expected 278 bp DNA band, which was visualized using agarose gel electrophoresis (Fig. 2). Thus, HRVA/C-F/HRVA/C-R2 was chosen for subsequent RT-RPA reactions.

**Fig. 3:**
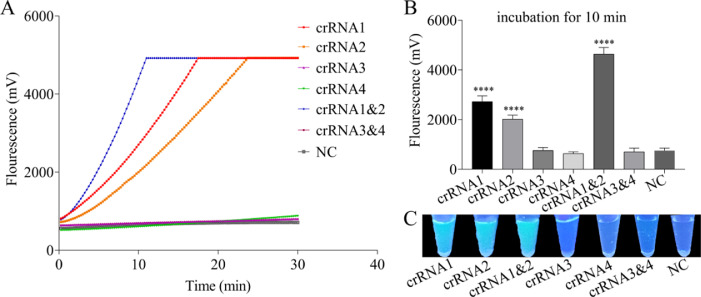
Screening the optimal crRNA using RT-RPA-Cas12a-mediated fluorescence assay with ssDNA FQ. (A) Representative real-time fluorescence kinetics of single or the combination of crRNA in the RT-RPA-Cas12a-mediated real-time fluorescence assay. (B) Fluorescence signals of single or the combination of crRNA obtained at 10 min in the RT-RPA-Cas12a-mediated end-point fluorescence assay. (C) Visualization of fluorescence signals of single or the combination of crRNA under a UV light illuminator at 20 min in the RT-RPA-Cas12a-mediated end-point fluorescence assay. The data were presented as mean ± SD (*n* = 3). NC, no crRNA control group.

Previous studies showed that crRNA plays a crucial role in affecting the trans-cleavage activity of Cas12a nuclease (Creutzburg et al., 2020; Zhang et al., 2018). Consequently, to obtain the optimal crRNA for subsequent experiments, four pairs of sequences of crRNA were designed based on the consensus sequences of VP4 for HRV-A and HRV-C. Then, each crRNA and each combination of crRNA in equal proportion were evaluated using the RT-RPA-Cas12a fluorescence assay, in which the final concentration of crRNA used was 80 nM, and the working concentration of Cas12a was set as 40 nM. As presented in Fig. 3A, a sharp increase in fluorescence signals was observed in the beginning 10 min for the combination of crRNA1 and crRNA2 in equal proportion with improved target cleavage efficiency, and leveled off over the following 20 min for crRNA1, crRNA2 and their combinations in equal proportion. In contrast, no detectable fluorescence signals were generated for poorly-performing crRNA3, crRNA4 and their combinations in equal proportion. Moreover, the end-point fluorescence signals were plotted in Fig. 3B. As displayed in Fig. 3B, compared with the single crRNA-mediated Cas12a detection system, the combination of crRNA1 and crRNA2 greatly generated the highest fluorescence intensity compared to other crRNA-treated groups when the target DNA concentration was equal (*p* < 0.001). Remarkably, as shown in Fig. 3C, distinct green fluorescent signals in the crRNA1, crRNA2 or their combination-treated group were observed under a UV light illuminator, compared with those in the addition of the crRNA3, crRNA4, or their combination-treated and untreated group. Similarly, the combination of crRNA1 and crRNA2 generated intense green fluorescences compared with other crRNA-treated groups.

**Fig. 4:**
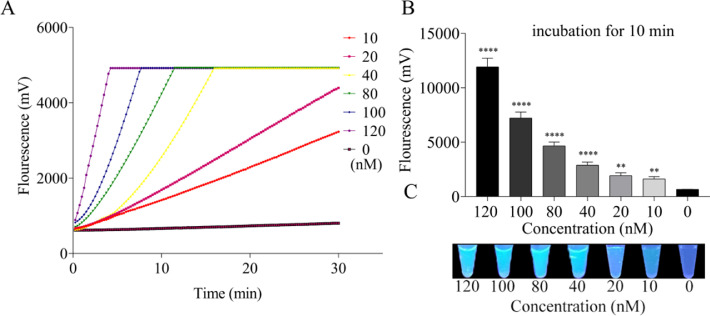
Screening the optimal crRNA concentration in the RT-RPA-Cas12a-mediated fluorescence assay with the combination of crRNA1 and crRNA2 in proportion at various concentrations (0, 10, 20, 40, 80, 100, 120 nM). (A) Representative real-time fluorescence kinetics of the combination of crRNA1 and crRNA2 in the RT-RPA-Cas12a-mediated real-time fluorescence assay. (B) Fluorescence signals of the combination of crRNA1 and crRNA2 obtained at 10 min in the RT-RPA-Cas12a-mediated end-point fluorescence assay. (C) Visualization of fluorescence signals of the combination of crRNA1 and crRNA2 under a UV light illuminator at 20 min using RT-RPA-Cas12a-mediated end-point fluorescence assay. The data were presented as mean ± SD (*n* = 3). NC, no crRNA control group.

To further investigate the effect of the concentration of the combination of crRNA1 and crRNA2 on the Cas12a-mediated trans-cleavage capacity, fluorescent signals were detected and compared in the same experimental condition. As demonstrated in Fig. 4A, in the presence of target standard RNA and ssDNA reporters, the fluorescent signals dramatically enhanced with the increase of crRNA concentration when all other reagents remain constant. Remarkably, the Cas12a reactions triggered by sufficient quantities of crRNA (≥80 nM) were completed within 10 min with the highly similar and identifiable slope, and the fluorescence kinetic curves plateaued thereafter. Though the combination of crRNA1 and crRNA2 at the final concentration of 120 nM generated the highest fluorescence intensity, 80 nM was selected as excess crRNA may lead to crRNA-independent cleavage. Therefore, the combination of crRNA1 and crRNA2 was chosen as the optimal crRNA, and 80 nM was applied in the following RT-RPA-Cas12a-mediated experiments. Moreover, the whole process can be completed within 50 min with high-efficiency.

### 3.2 Examination of specificity of the RT-RPA-Cas12a-mediated fluorescence assay

**Fig. 5:**
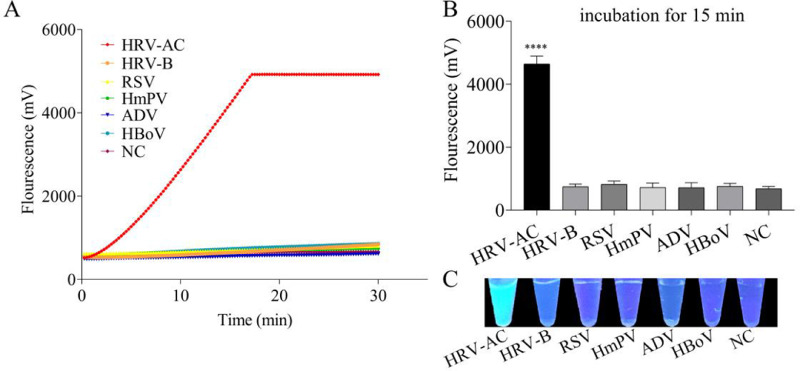
Specificity evaluation of RT-RPA-Cas12a-mediated real-time and end-point fluorescence assay, using various RNA or DNA samples from other respiratory viruses at the final concentration of 200 copies/μL, including standard RNA template of the consensus sequences for HRV-A and HRV-C (HRV-AC), human rhinoviruses B species (HRV-B), metapneumovirus (HmPV), adenovirus (ADV), bocavirus (HBoV), and respiratory syncytial virus (RSV), as the template of RT-RPA, respectively. (A) Specificity evaluation of RT-RPA-Cas12a-mediated real-time fluorescence assay with a fluorescence detector readout. (B) Results of the RPA-Cas12a-mediated end-point fluorescence assay at 15 min. (C) Specificity evaluation of RT-RPA-Cas12a-mediated end-point fluorescence assay with a naked-eye readout under a UV light illuminator. The data are presented as mean ± SD (*n* = 3). NC, no template control group.

Five other respiratory viral samples, including HmPV, ADV, HBoV, RSV, and HRV-B were employed as templates to investigate the specificity of the RT-RPA-Cas12a-mediated fluorescence assay. As shown in Fig. 5A, in the RT-RPA-Cas12a-mediated real-time fluorescence assay, no cross-reactivity was observed for other viral RNA or DNA samples with cycle-threshold values of < 30, where only standard RNA sample of the consensus sequences for HRV-A and HRV-C was found to generate remarkable fluorescence signals. Furthermore, the end-point fluorescence signals at 15 min were plotted in Fig. 5B, indicating the significant difference of fluorescence values between the target standard RNA positive group and other negative groups (*p<*0.05). Consistent with both real-time and end-point fluorescence detection of the RT-RPA-Cas12a-mediated system, the robust specificity of the RT-RPA-Cas12a-mediated fluorescence assay was observed by the naked eye under a UV light illuminator (Fig. 5C). Therefore, the validation of the RT-RPA-Cas12a-mediated fluorescence assay on the standard RNA sample displayed the robust specificity.

### 3.3 Examination of the sensitivity of the RT-RPA-Cas12a-mediated fluorescence assay

**Fig. 6:**
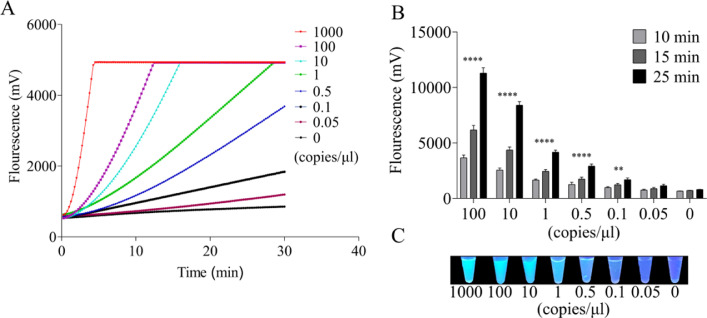
Evaluation of the sensitivity of the RT-RPA-Cas12a-mediated real-time and end-point fluorescence assay using a serial dilution of the standard RNA as the template. (A) Sensitivity examination of RT-RPA-Cas12a-mediated real-time fluorescence assay. (B) Sensitivity analysis of RT-RPA-Cas12a-mediated end-point fluorescence assay at different times. (C) Sensitivity analysis of RT-RPA-Cas12a-mediated end-point fluorescence assay at 20 min with a naked-eye readout under a UV light illuminator. The data are presented as mean ± SD (*n* = 3). NC, no template control group.

The sensitivity of the RT-RPA-Cas12a-mediated fluorescence or LFS assay was examined using a serial dilution of target standard RNA. As shown in Fig. 6A, in the RT-RPA-Cas12a-mediated real-time fluorescence assay, fluorescence signals generated by the Cas12a system were proportional to the final concentration of target standard RNA ranging from 1000 to 0.05 copies/μl, and the limit of detection (LOD) of the RT-RPA-Cas12a-mediated fluorescence assay was 0.1 copies/μl target standard RNA. Meanwhile, as shown in Fig. 6B, in the RT-RPA-Cas12a-mediated end-point fluorescence assay with a fluorescence detector readout, 0.1 copies/μl of target standard RNA was still able to generate a significant difference of fluorescence signals as compared with the control when the reaction time was more than 15 min. In contrast to both real-time and end-point fluorescence detection of the Cas12a system, only more than 0.5 copies/μl of target standard RNA was capable of producing the clear positive signal, which was easily visualized by the naked eye under a UV light illuminator, indicating that the LOD of the RT-RPA-Cas12a-mediated end-point fluorescence assay with a UV light illuminator readout was as low as 0.5 copies/μl (Fig. 6C).

### 3.4 Examination of the sensitivity and specificity of the RT-RPA-Cas12a-mediated LFS assay

**Fig. 7:**
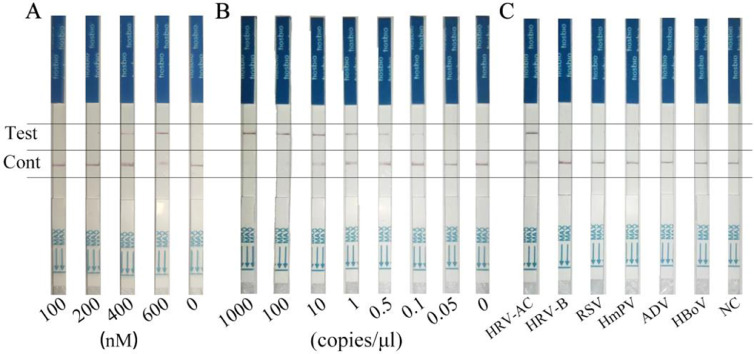
Establishing the RT-RPA-Cas12a-mediated LFS assay. (A) Screening the optimal crRNA concentration for the RT-RPA-Cas12a-mediated LFS assay using the combination of crRNA1 and crRNA2 in proportion. Various concentrations of the combination were used in the RT-RPA-Cas12a-mediated LFS assay. (B) Evaluation of the sensitivity of the RT-RPA-Cas12a-mediated LFS assay. A serial dilution of the standard RNA including the consensus sequences for HRV-A and HRV-C (HRV-AC) was employed as the template of RT-RPA. (C) Evaluation of the specificity of the RT-RPA-Cas12a-mediated LFS assay. Various RNA or DNA samples from respiratory viruses at the final concentration of 200 copies/μL, including standard RNA template of HRV-AC, human rhinoviruses B species (HRV-B), metapneumovirus (HmPV), adenovirus (ADV), bocavirus (HBoV), and respiratory syncytial virus (RSV), were employed as the template of RT-RPA, respectively. NC, no template control group.

For on-site detection, the RT-RPA-Cas12a-mediated assay was further integrated with LFS readouts to establish the RT-RPA-Cas12a-mediated LFS assay (Fig. 1A). To this end, the optimal concentration of the combination of crRNA1 and crRNA2 in equal proportion was firstly screened for strong, quantifiable signals in the RT-RPA-Cas12a-mediated LFS assay. As demonstrated in Fig. 7A, 600 nM of the combination effectively exhibited excellent trans-cleavage activity induced by Cas12a, with a clear band in the test line, and was chosen as the working concentration in RT-RPA-Cas12a-mediated LFS assay. Then, a serial dilution of target standard RNA was applied to examine the sensitivity of the RT-RPA-Cas12a-mediated LFS assay. As shown in Fig. 7B, only standard RNA sample produced a clear positive test band, implying that the LOD of the RT-RPA-Cas12a-mediated LFS assay was 0.1 copies/μl, which was consistent with that of the RT-RPA-Cas12a-mediated fluorescence assay with a fluorescence detector readout. We then tested the specificity of the RT-RPA-Cas12a-mediated LFS assay in terms of the detection of other viral samples with cycle-threshold values of <30, including HmPV, ADV, HBoV, RSV, and HRV-B. Fig. 7C indicated that only standard RNA sample exhibited a clear test band, and no cross-reactivity was observed for non-HRV-A or HRV-C samples. These results clearly validated the excellent specificity of the established RT-RPA-Cas12a-mediated LFS assay.

### 3.5 Clinical evaluation of the RT-RPA-Cas12a-mediated fluorescence or LFS assay for HRV-A or HRV-C detection

The RNA extraction of 80 samples was performed manually and automatically using magnetic bead-based extraction kit, respectively, of which the manual extraction of RNA can be prepared using a simple magnetic separator in resource-limited setting. Then, all RNA samples were tested and compared using both the RT-RPA-Cas12a-mediated fluorescence or LFS assay and qRT-PCR, where each RNA sample was tested in three independent experiments. As shown in Table 2, the positive predictive agreement of Cas12a-mediated fluorescence or LFS assay for HRV-A or HRV-C samples with qRT-PCR assay were 96.7% and 95%, respectively, whereas the negative predictive agreement of both assay was 100%. These results revealed that there was a high level of concordance between the detection results of HRV-A or HRV-C detected by established RT-RPA-Cas12a-mediated fluorescence or LFS assay and those of qRT-PCR. In this study, the workflow integrating robust amplification of the target nucleic acids with RT-RPA with specific detection of the amplicons with the Cas12a trans-cleavage activities, enables the higher concordance rate for RT-RPA-Cas12a-mediated detection assay compared to qRT-PCR.

**Table 2:** Comparison of RT-RPA-Cas12a-mediated fluorescence or lateral flow strip (LFS) assay and qRT-PCR of human rhinovirus species A or C in 80 clinical samples.

Assay	Number of samples		Detection coincidence rate with qRT-PCR for 60 positive samples	Detection coincidence rate with qRT-PCR for 20 negative samples
	Positive	Negative		
RT-RPA-Cas12a-mediated fluorescence	58	20	96.7%	100%
RT- RPA-Cas12a-mediated LFS	57	20	95%	
qRT-PCR	60	20		

## 4 Discussion

In humans, HRV, the main etiologic agent of the common cold, are responsible for significant morbidity, medical costs, and the loss of productivity in the workplace and school (Stobart et al., 2017; Fendrick et al., 2003). The significant health and economic burden caused by HRV highlights the dire need for improved access to early and rapid diagnosis. Especially, accurate, low-cost and rapid point-of-care (POC) diagnostics of HRV is crucial for identifying those at-risk for the illness associated with HRV, with the most frequently detected species, including HRV-A and HRV-C, accounting for the majority of acute respiratory infection (Giardina et al., 2022). Currently, qRT-PCR is the most routinely used method to detect HRV infections (Wisdom et al., 2009); however, qRT-PCR with the need of expensive equipment and skilled technicians, is not suitable to rapid POCT diagnostic tests in resource-limited setting.

In this study, to develop the RT-RPA-Cas12a-mediated fluorescence or LFS assay to detect HRV-A or HRV-C RNA viruses, a target sequence fragment of VP4 region that shared with 392 base pairs between HRV-A and HRV-C species was selected. Afterward, the target RNA for HRV-A and HRV-C as the template was prepared using the IVT method from the corresponding plasmids. In case of Cas12a, the cleavage efficiency varies significantly, depending on the structure of the crRNA (Creutzburg et al., 2020); nevertheless, the reliability of currently available tools for predicting the secondary structure of individual small RNA molecules is relatively low. Therefore, there is still no unified conclusion on how to realize the predictable success of crRNA design and target selection for the application of Cas12a nucleases, often resulting in designing 3–4 crRNAs for target a single gene. Thus, herein, four sequences of crRNA, including crRNA1, crRNA2, and crRNA3 and crRNA4, were designed for the VP4 region, and each crRNA can cover a 19 or 20 nucleotide region across the VP4 region. In contrast to Cas12a-mediated excellent collateral cleavage activity with crRNA1 and crRNA2, poorly-performing crRNA3 and crRNA4 failed to trigger the trans nonspecific ssDNA cleavage activity of Cas12a. This might be attributed to presence of a stable secondary structure in the crRNA3 and crRNA4. Moreover, similar to the previous report, compared with the single-crRNA-activated CRISPR/Cas12a system, the multiplex-crRNA-activated strategy enables an augmented rate of Cas12a-mediated collateral cleavage activity, as evidenced by the sharper upward slope of the reaction curve when the target RNA concentration was equal (Zeng et al., 2022). By screening out the optimal reaction parameters such as primer and crRNA, our established RT-RPA-Cas12a-mediated fluorescence or LFS assay can detect as low as 0.1 copies of either HRV-A or HRV-C RNA, which is 100 times more sensitive than that of a previous report of isothermal nucleic acid amplification-based detection method (RT-SIBA), with the sensitivity of as little as 10 copies of either HRV-A or HRV-B RNA (Kainulainen et al., 2019). Another advance of our method is the simplicity of the requirements for on-site detection instruments by combining the Cas12a tool and LFS to create a method, in which a rapid LFS-based readout with naked eye is a simple, straightforward and instrument-free biochemical analysis method.

To further examine the utility of established RT-RPA-Cas12a-mediated fluorescence or LFS assay on clinical samples, we tested crude manual and automated RNA extractions from 80 human respiratory samples that were previously routinely tested for HRV infection using qRT-PCR. The established RT-RPA-Cas12a-mediated real-time or end-point fluorescence assay unambiguously diagnosed HRV-A (32/31 agreement) and HRV-C (28/27 agreement) in each sample that was mixed with 200 genome copies/μL of HPV-B types, with high-consistency between the qRT-PCR-based detection and RT-RPA-Cas12a-mediated fluorescence signal within 50 min. A practical limitation for the wide-spread application of our established RT-RPA-Cas12a-mediated real-time fluorescence approach is the fact that a fluorescence spectroscopy is required. In this study, end-point fluorescence readouts were applied with excellent sensitivity and specificity using a simple portable instrument capable of detecting fluorescence signals. Also, to enhance utility and achieve visual detection, a LFS readout was employed to develop RT-RPA-Cas12a-mediated LFS assay, and achieved a LOD of 0.1 copies/μL. And the established RT-RPA-Cas12a-mediated LFS assay achieved highly consistent results with that of qRT-PCR method, with HRV-A (32/30 agreement) and HRV-C (28/27 agreement) 95% positive predictive agreement and 100% negative predictive agreement for HRV-A or HRV-C. For investigation on causes of false-negative results, two clinical samples that were tested false-negative by both RT-RPA-Cas12a-fluorescence and RT-RPA-Cas12a-LFS assays were amplified and sequenced. The results showed that there was two nucleotide sequence variations on the crRNA target site in the genomes of two clinical samples. This indicated that the mismatches between the target sequence and the crRNA might not be able to generate Cas12a-crRNA-target DNA complex, thereby causing false-negative results. In contrast, one sample that was tested false-negative in RT-RPA-Cas12a-LFS assay was amplified and sequenced, and there was no sequence variation on the crRNA target site. Further verification indicated that the false negative result might be attributed to the poor quality of commercial LFS employed. Altogether, these results present a new CRISPR-based diagnostic tool for HRV-A and/or HRV-C, similar to that developed for various pathogen DNA or RNA detection (Mohammad et al., 2022; Qian et al., 2021a, 2021b; Yin et al., 2021).

In sum, we established a new HRV-A and/or HRV-C molecular diagnostic assay that integrates RT-RPA amplification with CRISPR/Cas12a detection, with the result readout using a fluorescence detector or LFS. The established assay could be completed within 50 min without complex instruments and skilled technicians. The LOD of the RT-RPA-Cas12a-mediated real-time fluorescence or LFS assay could reach 0.1 copies/μl, and 0.5 copies/μl for the end-point fluorescence detection assay with a UV light illuminator readout. Taken together, these findings indicate that the RT-RPA-Cas12a-mediated fluorescence or LFS assay is a promising, and effective tool for routine and on-site detection method for HRV-A and/or HRV-C infections in resource-poor or constrained settings.

## Ethics approval and consent to participate

The use of clinical samples was approved by Ningbo Municipal Center for Disease Control and Prevention.

## Consent for publication

Not applicable.

## Availability of data and materials

All data generated or analyzed during this study are included in this manuscript.

## Funding

This work was supported partially by the Xi'an Science and Technology Plan Project (No. 22GXFW0007), the Science and Technology Program of State Administration of Market Supervision and Administration (No. 2021MK107), and Scientific Research Program Funded by 10.13039/501100009103Shaanxi Provincial Education Department (No. 22JC010).

## CRediT authorship contribution statement

**Weidong Qian:** Conceptualization, Methodology, Software, Writing – review & editing. **Xuefei Wang:** Data curation, Writing – original draft, Visualization, Investigation, Software, Validation. **Jie Huang:** Software, Validation. **Jian Liu:** . **Si Chen:** Supervision, Writing – review & editing. **Ting Wang:** Supervision, Conceptualization. **Dandan Zhang:** Conceptualization, Methodology, Software. **Yongdong Li:** Conceptualization, Methodology, Software, Writing – review & editing.

## Declaration of Competing Interest

The authors declare that they have no conflict of interest.

## Data Availability

No data was used for the research described in the article.

## References

[bib0026] Abudayyeh O.O., Gootenberg J.S., Konermann S., Joung J., Slaymaker I.M., Cox D.B., Shmakov S., Makarova K.S., Semenova E., Minakhin L., Severinov K., Regev A., Lander E.S., Koonin E.V., Zhang F. (2016). C2c2 is a single-component programmable RNA-guided rna-targeting CRISPR effector. Science.

[bib0021] Bates M., Zumla A. (2016). The development, evaluation and performance of molecular diagnostics for detection of mycobacterium tuberculosis. Expert Rev. Mol. Diagn..

[bib0029] Chen J.S., Ma E., Harrington L.B., Da Costa M., Tian X., Palefsky J.M., Doudna J.A. (2018). CRISPR-Cas12a target binding unleashes indiscriminate single-stranded dnase activity. Science.

[bib0033] Creutzburg S.C.A., Wu W.Y., Mohanraju P., Swartjes T., Alkan F., Gorodkin J., Staals R.H.J., van der Oost J. (2020). Good guide, bad guide: spacer sequence-dependent cleavage efficiency of Cas12a. Nucleic Acids Res..

[bib0036] Fendrick A.M., Monto A.S., Nightengale B., Sarnes M. (2003). The economic burden of non-influenza-related viral respiratory tract infection in the United States. Arch. Intern. Med..

[bib0007] Friedlander S.L., Busse W.W. (2005). The role of rhinovirus in asthma exacerbations. J. Allergy Clin. Immunol..

[bib0019] Garrido-Maestu A., Prado M. (2022). Naked-eye detection strategies coupled with isothermal nucleic acid amplification techniques for the detection of human pathogens. Compr. Rev. Food. Sci. Food Saf..

[bib0013] Giardina F.A.M., Piralla A., Ferrari G., Zavaglio F., Cassaniti I., Baldanti F. (2022). Molecular epidemiology of rhinovirus/enterovirus and their role on cause severe and prolonged infection in hospitalized patients. Microorganisms.

[bib0011] Golke P., Honemann M., Bergs S., Liebert U.G. (2021). Human rhinoviruses in adult patients in a tertiary care hospital in Germany: molecular epidemiology and clinical significance. Viruses.

[bib0027] Gootenberg J.S., Abudayyeh O.O., Kellner M.J., Joung J., Collins J.J., Zhang F. (2018). Multiplexed and portable nucleic acid detection platform with cas13, cas12a, and csm6. Science.

[bib0028] Harrington L.B., Burstein D., Chen J.S., Paez-Espino D., Ma E., Witte I.P., Cofsky J.C., Kyrpides N.C., Banfield J.F., Doudna J.A. (2018). Programmed DNA destruction by miniature CRISPR-Cas14 enzymes. Science.

[bib0002] Hayden F.G. (2004). Rhinovirus and the lower respiratory tract. Rev. Med. Virol..

[bib0005] Henquell C., Mirand A., Deusebis A.L., Regagnon C., Archimbaud C., Chambon M., Bailly J.L., Gourdon F., Hermet E., Dauphin J.B., Labbe A., Peigue-Lafeuille H. (2012). Prospective genotyping of human rhinoviruses in children and adults during the winter of 2009–2010. J. Clin. Virol..

[bib0006] Ison M.G., Hayden F.G., Kaiser L., Corey L., Boeckh M. (2003). Rhinovirus infections in hematopoietic stem cell transplant recipients with pneumonia. Clin. Infect. Dis..

[bib0001] Jacobs S.E., Lamson D.M., St George K., Walsh T.J. (2013). Human rhinoviruses. Clin. Microbiol. Rev..

[bib0015] Jia R., Lu L., Li S., Liu P., Xu M., Cao L., Su L., Xu J. (2022). Human rhinoviruses prevailed among children in the setting of wearing face masks in Shanghai. BMC Infect. Dis..

[bib0038] Kainulainen V., Elf S., Susi P., Maki M., Pitkaranta A., Koskinen J.O., Korpela R., Eboigbodin K.E. (2019). Detection of human rhinoviruses by reverse transcription strand invasion based amplification method (rt-siba). J. Virol. Methods.

[bib0004] Kiang D., Yagi S., Kantardjieff K.A., Kim E.J., Louie J.K., Schnurr D.P. (2007). Molecular characterization of a variant rhinovirus from an outbreak associated with uncommonly high mortality. J. Clin. Virol..

[bib0010] Kurai D., Saraya T., Ishii H., Takizawa H. (2013). Virus-induced exacerbations in asthma and copd. Front Microbiol..

[bib0012] Lamson D., Renwick N., Kapoor V., Liu Z., Palacios G., Ju J., Dean A., St George K., Briese T., Lipkin W.I. (2006). Masstag polymerase-chain-reaction detection of respiratory pathogens, including a new rhinovirus genotype, that caused influenza-like illness in New York state during 2004-2005. J. Infect. Dis..

[bib0009] Leigh R., Proud D. (2015). Virus-induced modulation of lower airway diseases: pathogenesis and pharmacologic approaches to treatment. Pharmacol. Ther..

[bib0018] Li J., Macdonald J., von Stetten F. (2018). Review: a comprehensive summary of a decade development of the recombinase polymerase amplification. Analyst.

[bib0008] McManus T.E., Marley A.M., Baxter N., Christie S.N., O'Neill H.J., Elborn J.S., Coyle P.V., Kidney J.C. (2008). Respiratory viral infection in exacerbations of copd. Respir. Med..

[bib0017] Milanoi S., Ongus J.R., Gachara G., Coldren R., Bulimo W. (2016). Serotype and genetic diversity of human rhinovirus strains that circulated in Kenya in 2008. Influ. Other Respir. Viruses.

[bib0030] Mohammad N., Katkam S.S., Wei Q. (2022). Recent advances in CRISPR-based biosensors for point-of-care pathogen detection. CRISPR J..

[bib0020] Owoicho O., Olwal C.O., Tettevi E.J., Atu B.O., Durugbo E.U. (2022). Loop-mediated isothermal amplification for candida species surveillance in under-resourced setting: a review of evidence. Expert Rev. Mol. Diagn..

[bib0031] Qian W., Huang J., Wang T., He X., Xu G., Li Y. (2021). Visual detection of human metapneumovirus using CRISPR-Cas12a diagnostics. Virus Res..

[bib0039] Qian W., Huang J., Wang X., Wang T., Li Y. (2021). CRISPR-Cas12a combined with reverse transcription recombinase polymerase amplification for sensitive and specific detection of human norovirus genotype gii.4. Virology.

[bib0016] Qin C., Liu J., Zhu W., Zeng M., Xu K., Ding J., Zhou H., Zhu J., Ke Y., Li L.Y., Sheng G., Li Z., Luo H., Jiang S., Chen K., Ding X., Meng H. (2022). One-pot visual detection of African swine fever virus using crispr-cas12a. Front .Vet. Sci..

[bib0035] Stobart C.C., Nosek J.M., Moore M.L. (2017). Rhinovirus biology, antigenic diversity, and advancements in the design of a human rhinovirus vaccine. Front Microbiol..

[bib0023] Tomita N., Mori Y., Kanda H., Notomi T. (2008). Loop-mediated isothermal amplification (lamp) of gene sequences and simple visual detection of products. Nat. Protoc..

[bib0024] Wang J.C., Yuan W.Z., Han Q.A., Wang J.F., Liu L.B. (2017). Reverse transcription recombinase polymerase amplification assay for the rapid detection of type 2 porcine reproductive and respiratory syndrome virus. J. Virol. Methods.

[bib0014] Watanabe A., Carraro E., Kamikawa J., Leal E., Granato C., Bellei N. (2010). Rhinovirus species and their clinical presentation among different risk groups of non-hospitalized patients. J. Med. Virol..

[bib0003] Winther B. (2011). Rhinovirus infections in the upper airway. Proc. Am. Thorac. Soc..

[bib0032] Wisdom A., Leitch E.C., Gaunt E., Harvala H., Simmonds P. (2009). Screening respiratory samples for detection of human rhinoviruses (hrvs) and enteroviruses: comprehensive vp4-vp2 typing reveals high incidence and genetic diversity of HRV species C. J. Clin. Microbiol..

[bib0025] Yan W.X., Hunnewell P., Alfonse L.E., Carte J.M., Keston-Smith E., Sothiselvam S., Garrity A.J., Chong S., Makarova K.S., Koonin E.V., Cheng D.R., Scott D.A. (2019). Functionally diverse type v CRISPR-Cas systems. Science.

[bib0040] Yin L., Man S., Ye S., Liu G., Ma L. (2021). CRISPR-Cas based virus detection: recent advances and perspectives. Biosens. Bioelectron..

[bib0037] Zeng M., Ke Y., Zhuang Z., Qin C., Li L.Y., Sheng G., Li Z., Meng H., Ding X. (2022). Harnessing multiplex CRRNA in the CRISPR/Cas12a system enables an amplification-free DNA diagnostic platform for ASFV detection. Anal. Chem..

[bib0034] Zhang C., Konermann S., Brideau N.J., Lotfy P., Wu X., Novick S.J., Strutzenberg T., Griffin P.R., Hsu P.D., Lyumkis D. (2018). Structural basis for the RNA-guided ribonuclease activity of CRISPR-Cas13d. Cell.

[bib0022] Zhang X., Li G., Chen G., Zhu N., Wu D., Wu Y., James T.D. (2021). Recent progresses and remaining challenges for the detection of Zika virus. Med. Res. Rev..

